# Validation of physics improvements for IMRT with a commercial treatment‐planning system

**DOI:** 10.1120/jacmp.v6i2.2083

**Published:** 2005-05-21

**Authors:** Patrick Cadman, Todd McNutt, Karl Bzdusek

**Affiliations:** ^1^ Medical Physics Department Saskatoon Cancer Centre 20 Campus Drive Saskatoon Saskatchewan Canada S7N 4H4; ^2^ Philips Medical Systems 6400 Enterprise Ln., #201 Madison Wisconsin 53719 U.S.A.

**Keywords:** radiation treatment planning, beam modeling, dosimetry, IMRT

## Abstract

A new Pinnacle 3D treatment‐planning system software release has recently become available (v7.4, Philips Radiation Oncology Systems, Milpitas, CA), which supports modeling of rounded multileaf collimator (MLC) leaf ends; it also includes a number of other software enhancements intended to improve the overall dose calculation accuracy. In this report, we provide a general discussion of the dose calculation algorithm and new beam‐modeling parameters. The accuracy of a diode dosimeter was established for measurement of MLC‐shaped beam profiles required by the new software version by comparison with film and ion chamber measurements in various regions of the field. The results suggest that a suitable diode or other small volume dosimeter with appropriate energy sensitivity should be used to obtain profiles for commissioning the planning system. Film should be used with caution, especially for larger field profile measurements. The dose calculation algorithm and modeling parameters chosen were validated through various test field measurements including a bar pattern, a strip pattern, and a clinical head and neck IMRT field. For the bar and strip patterns, the agreement between Pinnacle calculations and diode measurements was generally very good. These tests were helpful in establishing the new model parameter values, especially tongue‐and‐groove width, additional interleaf leakage, rounded leaf tip radius, and MLC transmission. For the clinical head and neck field, the comparison between Pinnacle and film measurements showed regions of approximately 2 cGy under‐ or overdose. However, the Pinnacle calculations agreed with diode measurements at all points to within 1 cGy or 1% of the maximum dose for the field (67 cGy). The greatest discrepancy between film and diode measurements for the clinical field (maximum of 2.8%) occurred in low‐dose regions in the central part of the field. The disagreement may be due to the overresponse of film to scattered radiation in the low‐dose regions, which have significant shielding by the MLCs.

PACS numbers: 87.53.Bn, 87.53.Dq

## I. INTRODUCTION

In a previous publication,^(1)^ validation of sequential intensity‐modulated radiation therapy (IMRT) with a commercial treatment‐planning system was reported (Pinnacle, Philips Radiation Oncology Systems, Milpitas, CA). The version of Pinnacle tested at that time, v6.2b, did not provide accurate modeling of the rounded leaf ends on the multileaf collimator (MLC) system tested (Millennium MLC, Varian Medical Systems, Palo Alto, CA). Dosimetric inaccuracies were reported and analyzed for calculations performed for sequential (step‐and‐shoot) IMRT that were directly attributable to the MLC leaf‐modeling strategy implemented by Pinnacle at that time.

A new software release has recently become available (v7.4) that supports modeling of rounded MLC leaf ends; the version also includes a number of other software enhancements intended to improve the overall accuracy of dose calculation. In this report, we provide a general discussion of the dose calculation algorithm and new beam‐modeling parameters. We then detail the commissioning and modeling process used to arrive at initial parameter values. Finally, we validate the dose calculation algorithm and modeling parameters chosen, by performing various validation measurements.

## II. MATERIALS AND METHODS

### A. Dose calculation algorithm and physics enhancements

The convolution superposition dose algorithm consists of three parts: creation of an incident energy fluence map for a beam, the computation of a 3D TERMA grid by projecting the incident energy fluence through the patient representation, and superposition of the TERMA with a dose deposition kernel to compute the dose in the patient.^(2^ ^–^ ^6)^ For IMRT, particular consideration is required for the modeling of the MLC characteristics and the scatter and output effects of small MLC fields in larger jaw settings. The following discussion summarizes the superposition algorithm identifying the improvements to more accurately support IMRT.

The incident energy fluence contains the modeling of the MLC and scattered radiation from the accelerator head. The incident energy fluence models both the shape of the radiation field as well as the calibration of the accelerator using the fluence/monitor unit (MU) formalism. The parameters of the incident energy fluence model for MLC‐defined fields include the following:

- separate *x* and *y* jaw transmissions
- width and height of the Gaussian head scatter source
- a radially symmetric arbitrarily shaped profile of the fluence to model the “horns” in the field
- *x* and *y* effective source size
- MLC parameters:
  - MLC transmission
  - rounded leaf tip radius
  - MLC leaf position offset as a function of the nominal MLC leaf position
  - tongue‐and‐groove width
  - additional interleaf leakage transmission

The evolution of the incident energy fluence begins with a uniform array large enough to encompass the beam. The “cone” generated from the radially symmetric arbitrary shaped profile is then extracted from the fluence array. Next, the jaw transmissions are applied to regions below the particular jaws.

**Figure 1:**
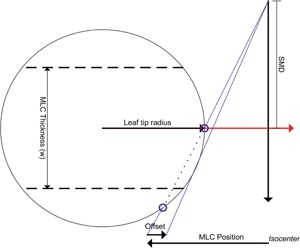
The geometry of the rounded leaf end and offset used to model the leaf tips of an MLC. The offset of the leaves applied by the accelerator is specified in distances at the isocenter for a range of nominal MLC leaf positions. This offset is applied during the generation in the incident energy fluence. SMD is the source to MLC distance.

The MLCs are modeled by creating an effective transmission array. This array is created to model the presence of the MLC leaf, the rounded leaf ends, and the tongue‐and‐groove effects. In regions below the full thickness of a leaf, the MLC transmission parameter is used. Below the leaf tip, the rounded leaf tip radius and the leaf position offset are used to generate the increase in transmission in the transition from the full thickness MLC leaf to the leaf tip. Figure 1 identifies the geometry, and the attenuation of the beam through the leaf tip is derived from the MLC transmission using the MLC thickness and the transmission to determine the effective attenuation coefficient for the MLC material. In regions where the tongue or groove is at the edge of the field, the transmission is calculated for one‐half a leaf thickness and applied over the tongue‐and‐groove width. In regions between two adjacent leaves, a specified additional interleaf leakage transmission is applied over the tongue‐and‐groove width. The final effective MLC transmission array is multiplied by the incident energy fluence array to incorporate the presence of the MLC.

**Figure 2:**
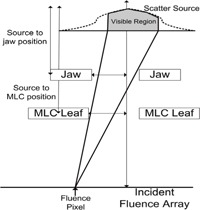
A graphical representation of the visible portion of the Gaussian head scatter source identifying how the head scatter is incorporated into the incident energy fluence in the presence of an MLC.

Scattered radiation from the head of the accelerator is modeled using a 2D Gaussian distribution at the position of the flattening filter. The scattered radiation is approximated by integrating over the “visible” portion of the Gaussian from each pixel in the incident energy fluence array. The “visible” portion is defined by the most limiting aperture shape based on the jaw and MLC positions as depicted in Fig. 2. As the aperture decreases in size, so does the head scatter contribution, thus modeling the decrease in head scatter for small MLC fields with larger jaw settings. Output factors for these fields are tabulated for the equivalent square defined by the jaws when the MLC does not replace one of the jaws. Using the Gaussian head scatter model allows the accurate prediction of the reduced head scatter when the fields are highly collimated by the MLC without requiring output factors to be derived from MLC shapes.

For step‐and‐shoot IMRT fields, the incident energy fluence for the beam is the weighted sum of the individual incident energy fluence arrays for each beam segment. The resultant incident energy fluence is then used to compute the TERMA and perform the superposition to obtain the dose distribution in the patient.

### B. Dosimeter accuracy

Dosimetry data required for commissioning the Pinnacle treatment‐planning system include beam profile measurements for both collimated and MLC‐shaped fields and output factor measurements as a function of field size for collimated fields. Various dosimeters are available for beam profile measurements, and each has advantages and disadvantages in terms of active volume, energy sensitivity, precision, usability, and overall accuracy. The version of Pinnacle treatment‐planning software tested in this report (v7.4) requires profile measurements for MLC‐shaped fields in addition to collimated fields. Accurate measurement of the MLC profiles is an essential starting point for commissioning of a treatment‐planning system for IMRT.^(1,^, ^7)^ Profiles must be measured with a dosimeter that has a small enough sensitive volume to achieve the necessary spatial resolution, especially in the high‐dose gradient regions at the MLC leaf edge. The response of various detectors in narrow photon beams, including a natural diamond detector, liquid ionization chamber, plastic scintillator and silicon diodes, has been compared.^(8)^

We established the accuracy of three dosimeters (ion chamber, diode, and film) by comparing profiles measured at a depth of 10 cm for square MLC‐shaped fields with sides of 3 cm, 10 cm, and 20 cm. An IC‐15 ion chamber (Wellhofer, Schwarzenbruck) was employed that has an inner diameter of 0.6 cm and an active volume of 0.13 cm^3^. The diode used was a photon field detector (PFD) (Scanditronix Medical AB, Uppsala, Sweden), which has an effective detection area of 0.025 cm^2^ and uses a tungsten/epoxy layer to provide energy compensation by filtering low‐energy photons. Ion chamber and diode profile measurements were performed using a Wellhofer water phantom system and the 6 MV beam from a Clinac 21EX LINAC (Varian Medical Systems, Palo Alto, CA) with a 120 leaf Millennium MLC. Kodak EDR2 film (Eastman Kodak, Rochester, NY) was irradiated in a polystyrene phantom at a depth of 10 cm. The film dosimetry results were scaled by a factor of 0.983 to account for the difference in radiological depth between polystyrene and water for the 6 MV beam to allow for comparison with ion chamber and diode measurements at the same depth (10 cm) in water. The scaling factor of 0.983 was arrived at by taking the average ratio of ion chamber measurements in the phantom and water at 10 cm depth for various small field sizes. The energy sensitivity of the diode and film was determined by comparison with the ion chamber measurements in the open part of the irradiated fields and under the leaves. The volume effect of the diode was evaluated by comparing the profiles in the penumbra region against film measurements.

EDR2 film was used for all film dosimetry measurements. Films were scanned with a 12‐bit Vidar film scanner (Vidar Systems Corp., Herndon, VA) and saved in .tiff file format. Dose comparison software was employed (DoseLab v3.05, created by Nathan Childress, Ph.D., and Isaac Rosen, Ph.D., University of Texas, M. D. Anderson Cancer Center, Houston, TX). The software provides a method for measuring a film sensitometric curve using a single sheet of film exposed with a two‐field step‐and‐shoot MLC delivery technique.^(9)^ Eight separate regions were irradiated on the EDR2 film with a maximum dose of 115 cGy using the same polystyrene phantom as for the film measurements. The absolute dose at the center of each of the regions was determined by using a parallel‐plane ion chamber calibrated under standard conditions. The DoseLab software was also used to generate dose difference maps and to extract profiles from the planar film dose distribution.

### C. Beam modeling using the physics enhancements and measured profiles

The version of Pinnacle software tested (v7.4) allows profiles shaped with the MLCs or collimators to be imported and modeled using the Photon Physics tool. Modeling of standard collimated and wedged fields in Pinnacle has been dealt with, in general, in a previous publication.^(10)^ Here, we will focus on the physics enhancements introduced with this software version intended to improve modeling of MLC‐shaped fields. The vendor suggests profile measurements for square MLC‐shaped fields of sides 2 cm, 5 cm, 10 cm, and 20 cm and asymmetric fields of 5 cm×20 cm and 20 cm×5 cm for the purpose of automodeling the beam and verification of beam parameters. We measured profiles perpendicular to the collimator rotation axis for MLC‐shaped square fields of sides 1 cm, 3 cm, 5 cm, 10 cm, and 20 cm with the collimator set to a 30 cm×30 cm field. The profiles for the 1 cm×1 cm field were obtained with film. All profiles were measured with the collimator oriented so that the MLC leaf travel would be perpendicular to the gun‐target (*y*) direction. All *y*‐profiles were measured through the central axis of collimator rotation. To avoid measuring the leakage between closed leaf pairs, the leaf ends were offset by 5 cm from the central axis. Profiles in the *x*‐direction (in the direction of leaf travel) were measured with an offset of 0.3 cm to avoid interleaf leakage between the MLC leaf sides.

The beam‐modeling parameters that were obtained during our initial Pinnacle commissioning, without the physics enhancements described in this paper, were used as a starting point for recommissioning with the physics enhancements. New parameter values are required in the MLC Editor portion of the Photon Physics tool. Chosen values for these MLC parameters are as follows: tongue‐and‐groove width: 0.1 cm; additional interleaf leakage transmission: 0.01; MLC leaf thickness: 6.76 cm; rounded leaf tip radius: 12.0 cm. Note that even though Varian documentation indicates that the MLC leaves have a radius of curvature of 8.0 cm, the leaf ends straighten out toward the top and bottom of the MLC, causing an apparent increase in the radius value. The choice of rounded leaf tip radius value will be discussed further in the strip pattern test results. The MLC leaf offset values correspond to the values in the MLCTABLE.TXT file supplied by Varian. Note that these parameters are not adjusted by the automodeling utility.

Pinnacle automodeler scripts may be run to help determine the optimal values for other beam parameters with the new MLC profile data. Specifically, the script labeled E_TuneInAllSections.OptSequence includes optimization of the jaw transmission, MLC transmission, and arbitrary fluence profile. The initial value of 0.020 for MLC transmission was determined from ion chamber measurements while commissioning the previous release of software, v6.2. The initial jaw transmission values chosen were 0.005 for each jaw, determined from the v6.2 automodeling. The default arbitrary fluence profile was chosen that linearly increases from the beam center and has 50 points. Note that the fluence grid resolution and phantom size used during automodeling will need to be set to define a relatively coarse dose calculation grid (perhaps 3 mm) so that dose calculation times are reasonable.

Although the adjustment of modeling parameters using the Photon Physics tool and direct examination of calculated and measured profiles may provide results that would be adequate for simple static MLC‐shaped fields, validation of the beam‐modeling parameters for IMRT requires more complex and comprehensive test fields as described next.

### D. Validation of beam‐modeling parameters using test fields

Three test fields were chosen for validation of the beam‐modeling parameters: a bar pattern, a strip pattern, and a clinical head and neck IMRT field. Film measurements were performed using EDR2 film placed at 10 cm depth in a polystyrene phantom at the isocenter plane. Corresponding point dose measurements were performed with the diode in a custom water tank at 10 cm. Diode scans were performed using the Wellhofer water phantom system with a source‐to‐surface distance of 100 cm and a depth of 10 cm. Diode profiles were rescaled to 100 source‐to‐probe distance. All dose calculations were performed using the Pinnacle Planar Dose Computation tool, which was configured to simulate the beam impinging on a water phantom with the same beam geometry as for the measurements. The resolution of the planar calculation grid was 1 mm for all calculation. Note that the main dose calculation grid (selectable in the main planning section) determines the TERMA grid resolution and should be set to match the planar dose computation grid.

**Figure 3:**
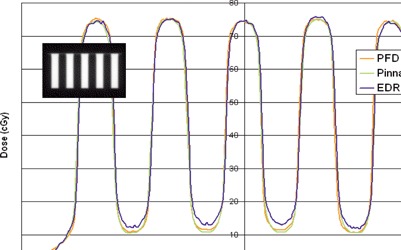
Bar pattern test profiles calculated by Pinnacle and measured with the PFD diode and EDR2 film. The bar pattern was composed of alternating open and closed regions of 2 cm height, formed by the MLC leaves in a 10 cm×20 cm collimated field (see insert). The profiles were obtained in the *y*‐direction, perpendicular to the direction of MLC leaf motion, and through the beam central axis.

The bar pattern was composed of alternating open and closed regions of 2 cm height, formed by the MLC leaves in a 10 cm×20 cm collimated field (see insert, Fig. 3). A total of 100 monitor units were used for the irradiation, giving a maximum dose of approximately 75 cGy at 10 cm depth. Profiles through the central axis in the *y*‐direction (i.e., perpendicular to the direction of leaf motion) were extracted from the planar film measurement and by scanning the beam with the diode connected to a UNIDOS electrometer (PTW Freiburg) in zero‐volt bias mode, in the Wellhofer water tank. The bar pattern test was used mainly to validate the tongue‐and‐groove leakage width parameter value as well as MLC transmission, primary and scatter source characteristics, and small field dosimetry in general.

**Figure 4:**
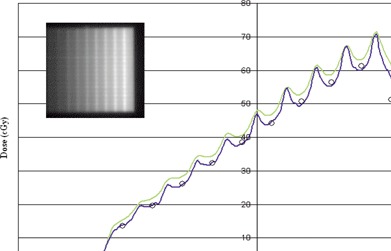
Strip pattern test profiles calculated by Pinnacle and measured with the PFD diode and EDR2 film. A strip pattern was obtained by irradiating 10 adjacent, 1 cm×10 cm MLC segments using segmental (step‐and‐shoot) delivery. The first segment has 7.28 MU, with each adjacent segment having an additional 7.28 MU, for a total of 400 MU for the field. (a) The MLC transmission value is 0.023; (b) the MLC transmission value is 0.018.

A strip pattern was obtained by irradiating 10 adjacent, 1 cm×10 cm MLC segments using segmental (step‐and‐shoot) delivery. The first segment has 7.28 MU delivered, with each adjacent segment having 7.28 MU in addition to the previous segment's MUs (see insert, Fig. 4). In this manner, a total of 400 MU were delivered, giving a maximum dose of approximately 72 cGy at 10 cm depth. Profiles in the *x*‐direction (i.e., in the direction of leaf motion) were obtained using film but were offset 0.3 mm from the central axis to avoid interleaf leakage. Corresponding point dose measurements were made every centimeter with the diode and UNIDOS electrometer in a custom water tank, for comparison. The strip pattern test was chosen mainly to validate the modeling of the rounded leaf ends as well as MLC transmission and small field dosimetry. A film profile obtained in the *y*‐direction (offset by 0.3 cm) was used to evaluate the leakage between the leaf sides and validate the selection of the additional interleaf leakage parameter in Pinnacle.

**Figure 5:**
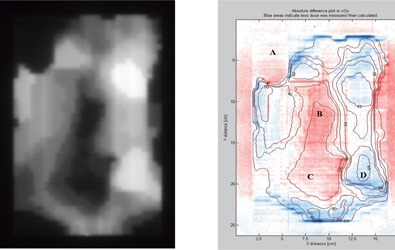
IMRT field test. (a) A grayscale dose image is provided to help identify the different dose regions; darker regions have lower dose. (b) The colors represent absolute dose difference values between Pinnacle calculations and film measurements in centigrays, according to the key on the right. Blue areas indicate that less was dose measured than calculated; red areas indicate that more dose was measured than calculated. The contains labeled isodose lines from the Pinnacle calculations. The letters indicate points in the field where diode measurements were made.

A clinical IMRT test field was chosen that was created for a nasopharyngeal patient treated at our center. The patient was planned with a simultaneous integrated boost technique with 66 Gy prescribed to the primary target with gross disease and 54 Gy to the regional lymph node areas. The field selected for analysis is the anterior field with collimator settings of 15.0 cm width and 22.5 cm length. The IMRT field was composed of 26 step‐and‐shoot segments. A grayscale representation of the dose calculated at a depth of 10 cm in a water phantom for this field is provided in (Fig. 5a). The IMRT field demonstrates large variability in dose delivered to various regions and probably represents an extreme in the amount of intensity modulation that might be encountered clinically. A dose difference image was obtained using the DoseLab software and displayed in terms of absolute dose difference in centigrays. A sample profile through both the high‐ and low‐dose regions was also obtained using DoseLab. Selected diode measurements were also performed at various points in the field to establish the accuracy of the film measurements and Pinnacle calculations.

## III. RESULTS AND DISCUSSION

### A. Dosimeter accuracy

**Figure 6:**
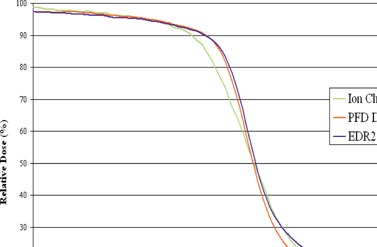
Profiles for a 10 cm×10 cm field defined by MLCs in the direction of leaf travel measured with an ion chamber, PFD diode, and EDR2 film. Note the overresponse under the MLC leaves with film but the agreement between film and diode in the 40% to 100 % dose region.

Profile measurements were made at a depth of 10 cm in water using the 6 MV beam for square MLC‐shaped field with sides of 3 cm, 10 cm, and 20 cm using the ion chamber and diode; Kodak EDR2 film was irradiated in a polystyrene phantom at the same depth. For the smallest field size (3 cm×3 cm), the agreement between all dosimeters was less than 1% at all depths within the open portion of the field and under the MLC leaves. However, in the penumbra region, the agreement between film and diode was excellent, with the ion chamber demonstrating a broadening in the penumbra width, due to the relatively large dimensions of the IC‐15 chamber. When the field size was increased to 10 cm×10 cm, the film measurements demonstrated an overresponse under the MLC leaves, but the agreement with the diode in the 40% to 100% dose region was still reasonable, as depicted in Fig. 6. The agreement between the diode and IC‐15 was reasonable in the central portion of the field and under the MLC. The results for the 20 cm×20 cm were essentially the same as for the 10 cm×10 cm field, in that the film showed an increased response outside the open portion of the field. The agreement between the diode and ion chamber for the three field sizes, except in the penumbra, indicates that its energy response is comparable to the ion chamber, and the agreement between the diode and film in the penumbra region indicates that the diode's spatial resolution is appropriate for MLC profile measurements.

These results imply that a suitable diode or other small‐volume dosimeter with appropriate energy sensitivity should be used to obtain profiles for commissioning the planning system. Film should be used with caution for this beam energy, especially for larger field sizes.

## B. Beam modeling using the physics enhancements and measured profiles

**Figure 7:**
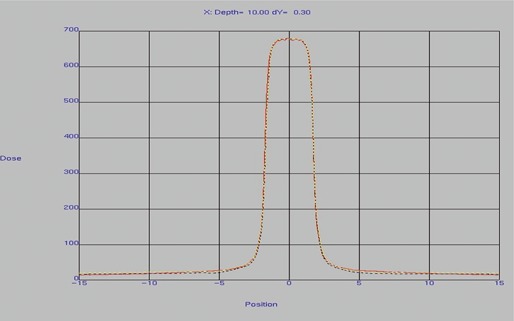
Modeled and measured profiles in the *x*‐direction (in the direction of leaf motion) for a 3 cm×3 cm MLC‐shaped field, as shown in the Pinnacle physics modeling tool. The red curve represents the diode measurements, the yellow dashed curve was calculated with an MLC transmission value of 0.023, and the black dashed curve was calculated with an MLC transmission value of 0.018.

After automodeling script E_TuneInAllSections.OptSequence, selected MLC profiles were recalculated with a 1 mm fluence grid before analysis. The automodeling results were very reasonable for both collimated and MLC profiles. Figure 7 depicts profiles in the *x*‐direction (in the direction of leaf motion) for the 3 cm×3 cm MLC‐shaped field. The red curve represents the diode measurements, the yellow dashed curve was calculated with an MLC transmission value of 0.023, and the black dashed curve was calculated with an MLC transmission value of 0.018. Note the excellent agreement for all curves in the central and penumbra regions. The curve with an MLC transmission value of 0.023 agrees better with diode measurements at a distance 5 cm from the beam center, and the curve with an MLC transmission value of 0.018 provides better agreement at 10 cm from the beam center. The choice of 12.0 cm for the MLC leaf tip radius gave the best overall results for the profiles. The choice of MLC transmission and MLC leaf tip radius parameters will be addressed further in the strip pattern test results.

### C. Test field validation results

#### C.1 Bar pattern test

The results for the bar pattern test are provided in Fig. 3. The profiles were obtained in the *y*‐direction, perpendicular to the direction of MLC leaf motion. The agreement between diode measurements and Pinnacle calculations indicates the proper choice for the tongue‐and‐groove width parameter and MLC transmission (0.018). Film measurements demonstrate a slight overresponse under the closed leaves.

#### C.2 Strip pattern test

The results for the strip pattern are provided in Fig. 4. The agreement between diode and film measurements is reasonable, except at a single point 4.5 cm from the central axis to the right, which may be due to a diode positioning (human) error. In (Fig. 4a), an MLC transmission value of 0.023 was used, and the calculated results are generally too high. When the transmission value was reduced to 0.018 ((Fig. 4b), the agreement between calculations and measurements greatly improved. Note also that the choice of MLC rounded leaf tip radius can have a significant effect on the strip pattern measurements. We have found that a radius value of 8 cm will produce a profile that is much like that presented in (Fig 4a). The choice of 0.018 for MLC transmission and 12.0 for the leaf tip radius gave the best overall results for the strip pattern test.

**Figure 8:**
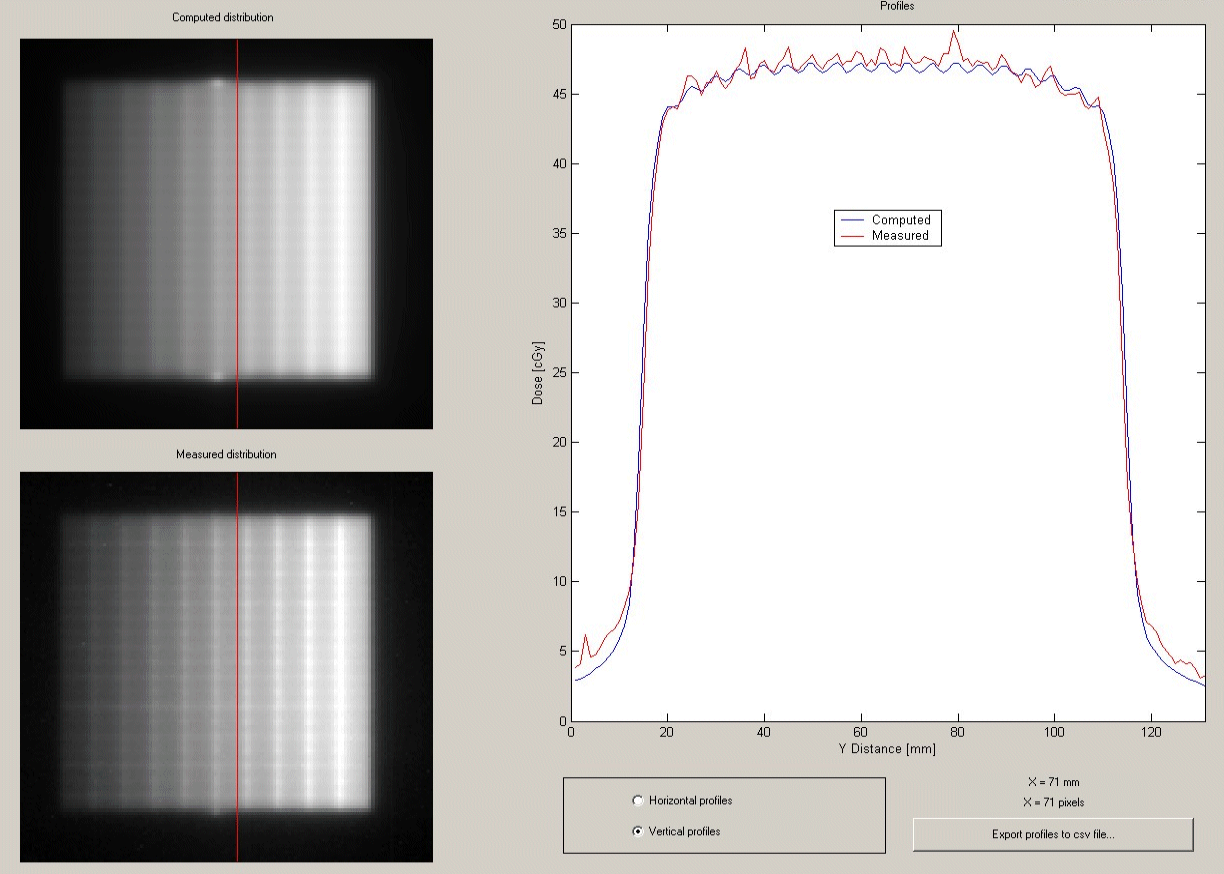
DoseLab profile comparison window showing profiles in the *y*‐direction for the strip test pattern. The Pinnacle‐computed distribution is shown in the upper‐left corner, and the measured distribution is shown in the lower‐left corner of the image. The red line indicates the location of the profile.

In Fig. 8 we show a profile in the *y‐*direction offset 3 mm from the central axis. This profile was used to evaluate the choice of additional interleaf leakage transmission parameter. Although there is considerable variability in the measured leakage, due to the mechanical tolerances of the MLC leaves themselves, an additional interleaf leakage transmission value of 0.01 seems to give an acceptable overall agreement.

#### C.3 Clinical IMRT field test

A dose difference map between Pinnacle calculations and film measurements for the clinical head and neck IMRT field is provided in (Fig. 5b). The colors in the map represent absolute dose difference values in centigrays, according to the key on the right of the figure. Blue areas indicate that less dose was measured than calculated; red areas indicate that more dose was measured than calculated. The figure also contains labeled isodose lines from the Pinnacle calculations, and a grayscale image of the Pinnacle dose is provided as an insert to help identify the different dose regions. Note that the high‐dose regions correspond to the bright areas in the Pinnacle planar dose image in (Fig. 5a), and the maximum dose is approximately 67 cGy. It was felt that the absolute dose difference was an appropriate way to validate the clinical IMRT field, since normalization to the dose maximum would have tended to underrepresent the dose differences in the low‐dose regions, which often correspond to the critical structures.

Generally, the absolute difference between Pinnacle and film measurements is less than 2 cGy (indicated by the white regions in the figure); however, there are some regions that indicate approximately a 2 cGy under‐ or overdose. In general, the dose measured in the high‐dose regions is less than calculated (blue), and the dose in the low‐dose regions is greater than calculated (red). Knowing that the EDR film can demonstrate an increased sensitivity in regions of low dose, as indicated by our bar test pattern results, we performed diode measurements at various points in the IMRT field to check the accuracy of the calculations and measurements. These points are labeled as follows: A, B, C, and D in (Fig. 5b). The Pinnacle calculation agreed with the diode measurements at all points to within 1 cGy or 1% of the maximum dose for the field (67 cGy). The film measurements agreed with the diode values to within 1% at all points except for points B and C, which were 2.8% and 2.1% greater than the diode measurements, respectively, when normalized to the field maximum. These points are in low‐dose regions in the central part of the field. The disagreement here may be due to the overresponse of film in low‐dose regions with significant shielding by the MLCs.

**Figure 9:**
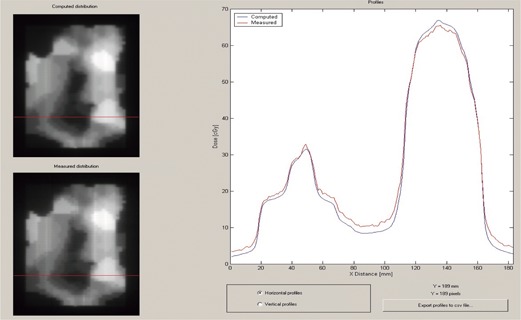
DoseLab profile comparison window showing profiles in the *x*‐direction (in the direction of leaf motion) for the IMRT field test pattern. The Pinnacle‐computed distribution is shown in the upper‐left corner, and the measured distribution is shown in the lower‐left corner of the image. The red line indicates the location of the profile.

A DoseLab profile comparison for the IMRT field is shown in Fig. 9. The profile is in the *x*‐direction and passes through the approximate location of points C and D. Note that the agreement of the profiles is reasonable, especially in the high gradient regions, although there is a slight overestimate by Pinnacle in the high‐dose region and an underestimate in the low‐dose region. As suggested above, the film measurements in the low‐dose regions may be high due to the increased sensitivity in this region. If this were so, the overall discrepancy between the calculated and true values would be reduced.

## IV. CONCLUSION

A new Pinnacle software release has recently become available (v7.4) that supports modeling of rounded MLC leaf ends and includes a number of other software enhancements intended to improve the overall dose calculation accuracy. In this report, we have provided a general discussion of the dose calculation algorithm and new beam‐modeling parameters. The accuracy of a diode dosimeter has been established for measurement of MLC‐shaped beam profiles required by the new software version. The dose calculation algorithm and modeling parameters chosen were validated through various test field measurements, including a bar pattern, a strip pattern, and a clinical head and neck IMRT field. The agreement between Pinnacle and measurements for these fields indicates a significant improvement in accuracy due to the physics modeling enhancements. Our results also reveal that the accuracy of EDR2 film for use as a dosimeter for IMRT fields may be limited due to its spectral response.
